# Biochemical analysis of *Komagataella phaffii* oxidative folding proposes novel regulatory mechanisms of disulfide bond formation in yeast

**DOI:** 10.1038/s41598-023-41375-z

**Published:** 2023-08-31

**Authors:** Arianna Palma, Lukas A. Rettenbacher, Antti Moilanen, Mirva Saaranen, Christian Pacheco-Martinez, Brigitte Gasser, Lloyd Ruddock

**Affiliations:** 1 Department of Biotechnology, University of Natural Resources and Life Sciences, Vienna, Austria; 2 Austrian Centre of Industrial Biotechnology, Vienna, Austria; 3 School of Biosciences, University of Kent, Canterbury, UK; 4 Faculty of Biochemistry and Molecular Medicine, University of Oulu, Oulu, Finland

**Keywords:** Enzyme mechanisms, Protein folding, Endoplasmic reticulum

## Abstract

Oxidative protein folding in the endoplasmic reticulum (ER) is driven mainly by protein disulfide isomerase PDI and oxidoreductin Ero1. Their activity is tightly regulated and interconnected with the unfolded protein response (UPR). The mechanisms of disulfide bond formation have mainly been studied in human or in the yeast *Saccharomyces cerevisiae.* Here we analyze the kinetics of disulfide bond formation in the non-conventional yeast *Komagataella phaffii*, a common host for the production of recombinant secretory proteins. Surprisingly, we found significant differences with both the human and *S. cerevisiae* systems. Specifically, we report an inactive disulfide linked complex formed by *K. phaffii* Ero1 and Pdi1, similarly to the human orthologs, but not described in yeast before. Furthermore, we show how the interaction between *K. phaffii* Pdi1 and Ero1 is unaffected by the introduction of unfolded substrate into the system. This is drastically opposed to the previously observed behavior of the human pathway, suggesting a different regulation of the UPR and/or possibly different interaction mechanics between *K. phaffii* Pdi1 and Ero1.

## Introduction

Disulfide bond formation is one of the most common post-translational modifications found in proteins. In eukaryotes, disulfide bonds are formed in the endoplasmic reticulum (ER), a naturally oxidizing compartment^1,2^. This organelle hosts cross-kingdom conserved folding factors specialized for cysteine oxidation: the protein disulfide isomerase (PDI) family, of which PDI is the most common representative, and the ER oxidoreductin 1 (Ero1)^3–5^. PDI comprises of four thioredoxin-like domains (*a b b’ a’*), of which *a* and *a’* harbor a CGHC active-site sequence motif, while Ero1 contains two active site cysteine pairs (one shuttle disulfide and one inner active site). These two factors work synergistically to catalyze disulfide bonds through redox reactions. During catalysis, PDI oxidizes free thiols in nascent proteins through the active site on its *a’* domain, which in turn gets reduced. Ero1 is dedicated to the recycling of specific PDI family members through its shuttle disulfide (Cys94-99 of human Ero1α, or Cys100-105 of *Saccharomyces cerevisiae* Ero1). The reduced shuttle disulfide of Ero1 gets reoxidized through internal thiol-disulfide exchange with its active site, which transfers the reducing equivalents to a flavin cofactor and ultimately to molecular oxygen, thereby generating hydrogen peroxide. Additionally, PDI in its reduced state can rearrange incorrectly linked disulfides through its isomerase activity^6,7^. Unregulated activity of Ero1 could cause both the hyperoxidation of PDI, thus inhibiting isomerization and hence native disulfide formation, and the release of excessive oxygen radicals with subsequent cytotoxic effects^8,9^. To protect against such stresses, Ero1 is tightly tuned, mainly by the formation of regulatory intramolecular disulfides. Both oxidation and reduction of the regulatory disulfides were proposed to be performed by PDI, but the detailed mechanism is still not fully understood^10–12^. As an additional level of regulation, both α and β isoforms of human Ero1 have been shown to form an inactive covalent complex with human PDI (hPDI)^13^. During catalysis and regulatory inhibition, hPDI and Ero1 interact through the *b’* domain on hPDI and a protruding β-hairpin on Ero1. This structural feature is particularly developed on human Ero1α, while the β-hairpin of Ero1 in the yeast *S. cerevisiae* is still present but less protruding and was suggested being not as crucial in the interaction^14,15^.

Oxidative folding is also intertwined with quality control pathways avoiding the accumulation of unfolded proteins, which are generally referred to as the unfolded protein response (UPR)^16,17^. When misfolded proteins start accumulating in the ER, the folding capacity is initially upregulated by increased expression of chaperones^18,19^. If this is insufficient, the ER-associated degradation (ERAD) is activated^20,21^. It was recently shown how unfolded substrates can act as competitors for hPDI interacting with Ero1α by binding to the *b’* domain^22^. This mechanism allows client proteins to remain unfolded and reduced, two necessary requirements to enter the degradation pathway and to avoid futile redox cycles^23,24^.

Processes such as recombinant protein production hold disulfide bond formation as one of the most relevant bottlenecks. Yeasts such as *S. cerevisiae* and *Komagataella phaffii* (syn *Pichia pastoris)* are among the most important microbial production hosts and are employed to commercially produce disulfide-bonded proteins^25–27^. *K. phaffii* has been reported to have higher secretory capacity and production titers of more than 10 g/L of nanobodies or 3 g/L insulin have been obtained in fed-batch processes^28,29^. Recently, the first full length monoclonal antibody produced in *K. phaffii* was approved by the FDA^30^, and several antibody-derived molecules such as single-chain fragments and single-domain antibodies (vHH) are in the pipeline. Despite the importance of Ero1 and PDI, not much is known about their regulation and catalysis in yeasts, except for *S. cerevisiae.* For most other yeasts, including *K. phaffii,* Ero1 and Pdi1 have remained classified to date only as genes predicted by homology.

In this study, we analyze in detail the oxygen consumption kinetics of *K. phaffii* Ero1, as a representative of the fungal kingdom with relevant industrial applications in the biopharmaceutical field. We compare differences and similarities to so far reported data from human and *S. cerevisiae* and show how *K. phaffii* Ero1 and Pdi1 present hybrid characteristics between the yeast and the human system.

## Results

### Disulfide mapping and expression analysis of *K. phaffii* Ero1

**Figure 1:**
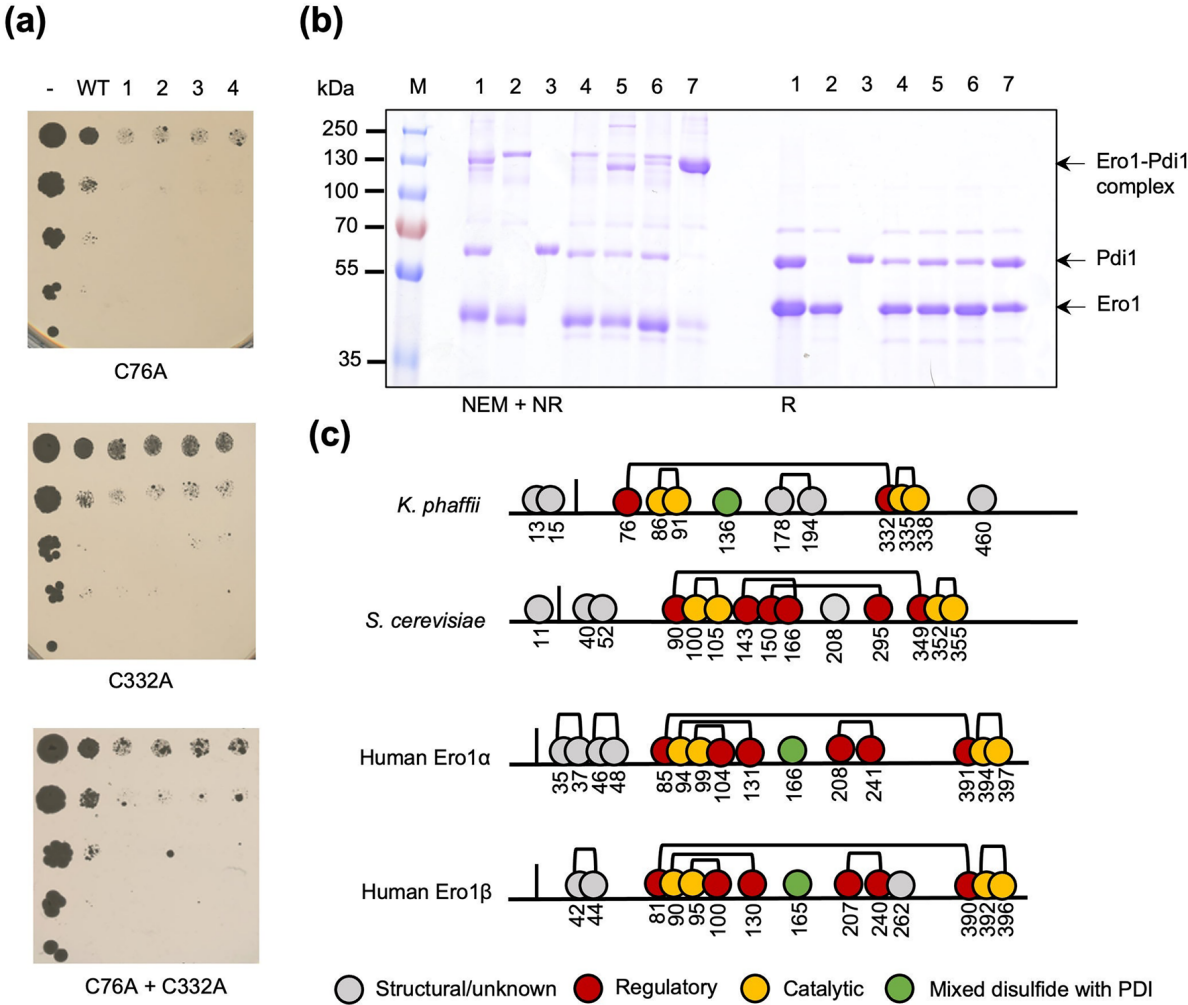
(**a**) Spotting assay of diluted *S. cerevisiae* BY4741 *Ire1*Δ overexpressing *K. phaffii* Ero1^C76A^ (C76A), Ero1^C332A^ (C332A) and in combination. Results obtained with the other four phenotypically neutral Cys/Ala mutations can be found in Supplementary Fig. S2. WT = wild-type Ero1,- = empty vector control, 1–4 = four independent clones overexpressing mutant Ero1; (**b**) SDS-PAGE showing small scale expression and IMAC purification of: (1) Co-expression of wild-type Ero1 and Pdi1, (2) Co-expression of Ero1^C136A^ and wild-type Pdi1, (3) wild-type Pdi1, 4() wild-type Ero1 and Pdi1^C62A^, (5) wild-type Ero1 and Pdi1^C65A^, (6) wild-type Ero1 and Pdi1^C404A^, (7) wild-type Ero1 and Pdi1^C407A^. In all cases, except for wild-type Pdi1 expressed alone, Ero1 was co-expressed with Pdi1 on the same construct and with the *K. phaffii* Pdi1 customized CyDisCo vector. On the left side of the gel, NEM-blocked non-reduced samples are shown, while, on the right side, reduced samples are shown in the same order. An uncropped image of the gel is provided in Supplementary Fig. S4; (**c**) proposed disulfide pattern of *K. phaffii* Ero1, in comparison to the previously identified patterns of *S. cerevisiae* Ero1 and the human Ero1α/β paralogs.

*K. phaffii* Ero1 is encoded by PP7435_Chr1-0304. It contains 12 cysteines, two of which are in its predicted signal peptide. Of the remaining ten in the mature protein, the pairs Cys86-Cys91 and Cys335-Cys338 are likely to represent the shuttle disulfide and the active site residues, as they are highly conserved in sequence alignments with the characterized human homologs and *S. cerevisiae* Ero1^7,31^ (Supplementary Fig. S1). In contrast, the positions of the other cysteines in *K. phaffii* Ero1 are not shared with the human and *S. cerevisiae* orthologs, so their structural or functional roles are less clear. Thus, we set out to identify the regulatory residues. Rather than develop and validate a *K. phaffii* based assay, our initial approach was to use a growth-based assay previously used for the same purpose in the yeast *S. cerevisiae*^7^. This assay is based on the toxicity of uncontrolled release of hydrogen peroxide by hyperactive Ero1 upon mutation of either or both cysteines forming a regulatory disulfide. The six cysteines of *K. phaffii* Ero1 remaining unmapped were singularly replaced by alanines and overexpressed in a *S. cerevisiae* strain defective in the UPR regulator Ire1, which is highly susceptible to redox stress^9^. Growth impairment compared to *ire1*Δ overexpressing wild type *K. phaffii* Ero1 could be observed only for the mutants Ero1^C76A^ and Ero1^C332A^ or the double mutant Ero1^C76A/C332A^ (Fig. 1a and Supplementary Fig. S2). This implies that the experimental approach was successful and that Cys76 and Cys332 are involved in forming one long-range regulatory disulfide bond whose removal generates a hyperactive variant.

Ero1 has often been studied in vitro as a monomer that transiently interacts with PDI. With the human paralogues, this approach was recently reconsidered in favor of the formation of an inactive heterodimeric Ero1-PDI complex, which interacts with exogenous reduced hPDI and leads to its activation^13^. This showed how hPDI acts to Ero1 both as a regulatory and catalytic partner. The regulatory complex was determined to be a covalent heterodimer and involves Cys166/165 of human Ero1α/β together with Cys397 of the hPDI *a’* domain^13^. Cys136 of *K. phaffii* Ero1 aligns to Cys166/165 in Ero1α/β (Supplementary Fig. S1), suggesting it could play the same role. This alignment matched also at the structural level, when we aligned the *K. phaffii* Ero1 structure predicted by AlphaFold^32,33^ with the crystal structure of Ero1α (Supplementary Fig. S3), indicating that *K. phaffii* Ero1 might share similarities with its human homolog.

Moilanen et al. could successfully isolate the human complex from the cytoplasm of an *Escherichia coli* CyDisCo strain without the need of extra in vitro steps to tackle redox inhomogeneity. This strain has been reported as a successful cell factory for a very diverse array of disulfide bonded proteins^13,22^. We therefore employed it for the expression of *K. phaffii* Ero1 and Pdi1*.* With a similar approach to previous purification methods reported for *S. cerevisiae* Ero1^9,34,35^, *K. phaffii* Ero1 was expressed without the amphipathic C-terminus, which is involved in localization to the inner membrane of the ER^35^. Classical CyDisCo, expressing hPDI as an isomerase and *S. cerevisiae* Erv1 as an oxidase, failed to deliver a redox homogeneous product for *K. phaffii* Ero1. We hypothesized that this was due to differences between hPDI and Pdi1. The CyDisCo vector was therefore customized with *K. phaffii* Pdi1 replacing hPDI. This resulted in the successful production of Ero1 in good yields and 1:1 co-purification of both wild-type Ero1 (45 kDa) and Pdi1 (56 kDa) was observed (Fig. 1b), as per the human proteins^13^.

To verify whether coelution depended on Cys136 of Ero1 forming a mixed disulfide with Pdi1, Cys136 was mutated into alanine. This resulted in the loss of Pdi1 coelution (Fig. 1b, lane 2). Therefore, assuming Cys136 of *K. phaffii* Ero1 is functionally homologous to Cys166/165 of human Ero1α/β, we set out to identify which residue on Pdi1 were involved in the complex-forming disulfide bond.

*K. phaffii* Pdi1 has two conserved active sites, one in its *a* domain (Cys62-Cys65) and the other in its *a’* domain (Cys404-Cys407). We hypothesized that if one of the active site cysteines participates in the mixed disulfide, the neighboring free thiol of the same domain could spontaneously perform a nucleophilic attack on the bond and resolve the complex. This would lead to an equilibrium between the Ero1-Pdi1 heterodimeric and non-covalent states. Therefore, removing the neighboring cysteine to the cross-linking disulfide should shift the redox balance towards the stable heterodimer. To test this hypothesis, we mutated singularly all four catalytic cysteines of *K. phaffii* Pdi1. Small-scale expression and IMAC of the complex where Cys407 was mutated into alanine allowed the visualization of the heterodimer as the main form under NEM trapping conditions on SDS-PAGE (Fig. 1b, lane 7). This suggested Cys407, as neighboring reactive residue to Cys404, causes resolution of the complex after denaturation in SDS. Therefore, we propose Cys136 of *K. phaffii* Ero1 is engaging in a complex with Cys404 of *K. phaffii* Pdi1, analogously to the interactions in the human counterparts (Fig. 1c).

### Folding quality of *K. phaffii* Ero1

**Figure 2:**
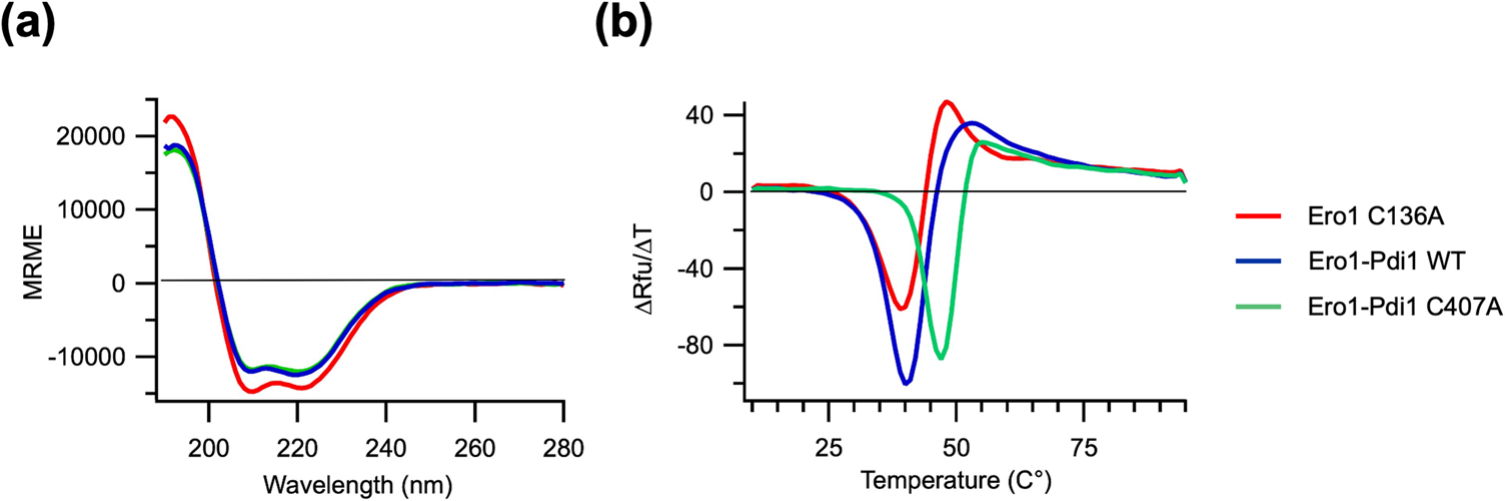
(**a**) Mean residue molar ellipticity (MRME) spectra of monomeric Ero1^C136A^, the wild-type Ero1-Pdi1 complex and the Ero1-Pdi1^C407A^ complex; (**b**) RFU derivative curves over temperature for monomeric Ero1^C136A^, the wild-type Ero1-Pdi1 and the Ero1-Pdi1^C407A^ complex.

Since the Ero1-Pdi1 complex (wild type and Cys407Ala mutant of Pdi1) and Ero1^C136A^ were expressed and purified from a bacterial host, their biophysical properties were assessed to examine product quality. It was observed that *K. phaffii* Ero1 lost yellow color during purification, consistent with a loss of bound FAD^+^. To reverse this, the protein was reflavinated by incubation with a 1:2 molar excess of FAD^+^ prior to the final purification step. After reflavination and size exclusion chromatography, the purified Ero1-Pdi1 complex had an average of 0.93 ± 0.05 FAD^+^ molecules per complex, while monomeric Ero1^C136A^ had an average of 0.85 ± 0.04. Circular dichroism scans were performed to evaluate regular secondary structure (Fig. 2a). All proteins showed a predominantly α-helical spectrum, as did Pdi1 (Supplementary Fig. S5). Molar ellipticity values were unaffected by the Cys407Ala mutation on Pdi1 in the complex (Fig. 2a) or by reflavination (Supplementary Fig. S5). Thermostability scans showed single transitions in all denaturation profiles. It was observed that both monomeric Ero1^C136A^ and the wild-type complex peaked at 40° C, similarly to the T_m_ of monomeric Pdi1 (Supplementary Fig. S6), while the Cys407Ala mutant, which traps the Ero1-Pdi1 complex in the heterodimeric state, was significantly more thermostable (by approximately 7 °C) (Fig. 2b). Reflavination did not affect thermostability (Supplementary Fig. S6).

Redox homogeneity was confirmed by reversed-phase liquid chromatography mass spectroscopy. In the absence of NEM, the wild-type disulfide-linked complex was observed as a redox homogeneous product. The average mass was consistent with the predicted disulfide bond distribution in Ero1 and the Cys136-Cys404 intermolecular disulfide (Supplementary Table S1). In contrast, NEM treatment resulted only in the detection of monomeric Ero1 and Pdi1 (as preliminarily suggested by the SDS-PAGE patterns in Fig. 1a).

### Kinetic analysis of *K. phaffii* Ero1-Pdi1 complex

As no previous studies have been carried out on *K. phaffii* Ero1, we aimed for detailed characterization of our in vivo folded Pdi1-Ero1 complex. To this end we adopted a similar set-up for Clark electrode-based oxygen consumption measurements as Baker et al*.*^36^ and Moilanen et al*.*,^13^ where Ero1 utilizes dissolved oxygen to oxidize Pdi, which in turn oxidizes GSH to GSSG and this is then reduced by glutathione reductase.

**Figure 3:**
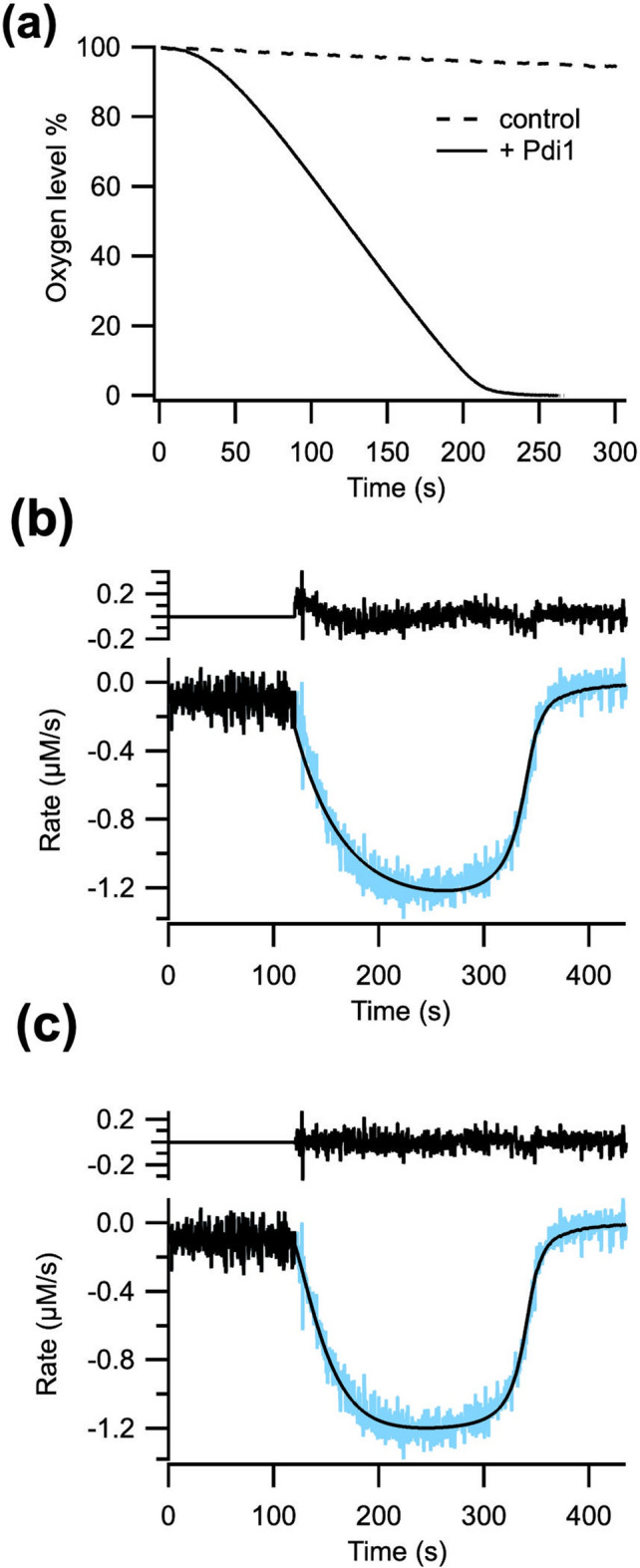
(**a**) Representative oxygen consumption trace of *K. phaffii* wild-type Ero1-Pdi1 complex in combination with tenfold exogenous *K. phaffii* Pdi1 (solid line); Control reaction with monomeric Ero1^C136A^ without supplied Pdi1 (dashed line); Differentiated oxygen consumption trace of wild-type Ero1-Pdi1 complex fitted to a single step- (**b**) and double step-activation model (**c**). Residuals are shown above the fitted traces.

Three distinct kinetic phases were observed: an initial lag phase, followed by a linear trend of maximal rate (V_max_) and a fast decline at very low oxygen concentrations (Fig. 3a). The non-catalyzed control performed with Ero1^C136A^ in the absence of Pdi1 showed no activity (Fig. 3a). The derivative of the oxygen consumption trace as a function of time and [O_2_] was then fitted into a model that combined either a one- or two-step activation process and steady-state Michaelis–Menten enzyme kinetics, with a Hill coefficient for oxygen, as previously used for the human enzymes^13^. As *K. phaffii* Ero1 appears to have only one intramolecular regulatory disulfide, we initially hypothesized a simpler activation process compared to the human system, which is characterized by a long biphasic activation^13^. However, a kinetic fit with only one activation step gave non-random residuals, suggesting a more complex mechanism, which was confirmed by adopting a two-step function (Fig. 3b,c). With this scheme of analysis, we were able to obtain the values for both activation rate constants, V_max,_ Km_[O2]_ and Hill coefficient for the Ero1-Pdi1 complex and monomeric Ero1^C136A^ (Table 1).

**Table 1:** Oxygen consumption kinetic parameters for 1 μM Ero1-pdi1 wild-type, Ero1-pdi1^C407A^ and monomeric Ero1^C136A^ in combination with tenfold external Pdi1. K_b_ was not detected for Ero1^C136A^.

Enzyme (1 µM)	Activation rate constants		Activation t_1/2_ (s)	K_cat_ (s^−1^)	K_M_ (µM)	Hill coeff
	K_a_ (s^−1^)	K_b_ (s^−1^)				
Ero1-Pdi WT	0.09 ± 0.01	0.03 ± 0.0040	32.33 ± 1.15	1.28 ± 0.06	13.65 ± 1.81	2.36 ± 0.31
Ero1-Pdi C407A	0.07 ± 0.01	0.01 ± 0.00010	95.0 ± 3.46	0.93 ± 0.03	9.63 ± 0.92	2.16 ± 0.15
Ero1 C136A	0.09 ± 0.005	–	7.89 ± 0.46	0.69 ± 0.03	13.07 ± 1.45	1.26 ± 0.19

**Figure 4:**
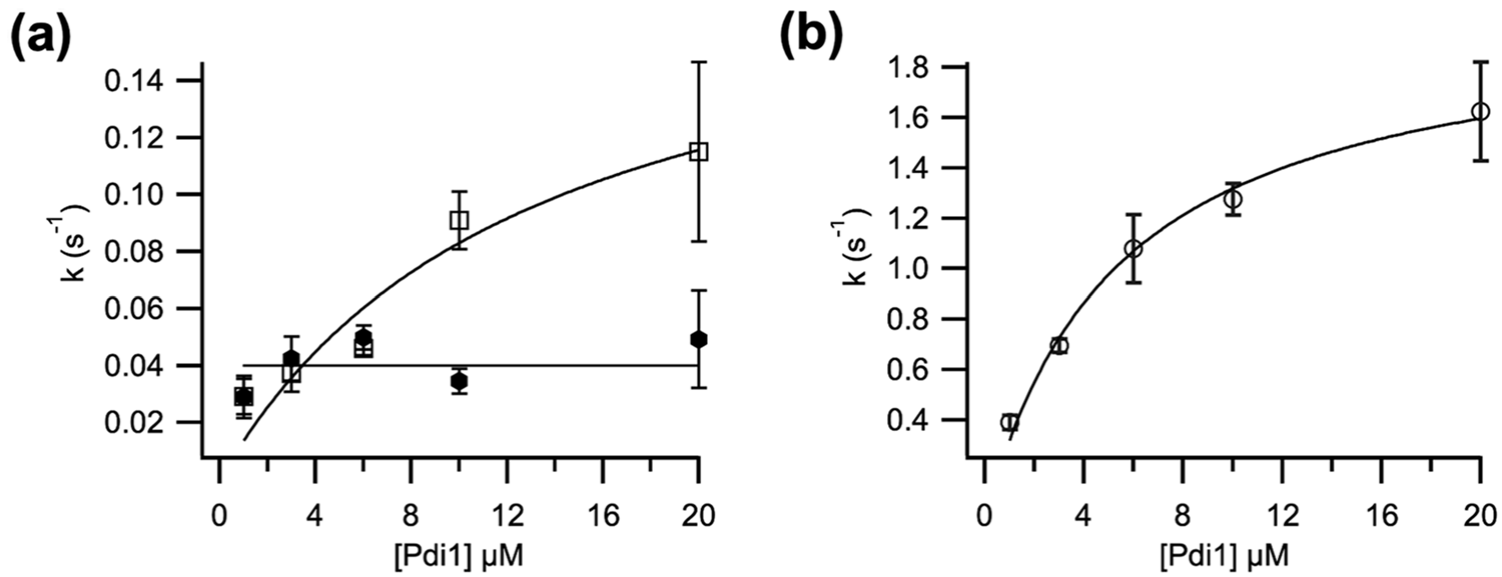
(**a**) Titration effect of supplied Pdi1 on first (open squares) and second (closed hexagons) activation rates, and (**b**) on K_cat_ for the wild-type complex. n = 3–5, data represented as average and standard deviation. Data was fitted to the classical Michaelis–Menten model.

We then proceeded to investigate the activation phase of the reaction. First a [Pdi1] titration was performed (Fig. 4a). The results showed one of the activation steps was dependent on [Pdi1], while the other was independent. In addition, it allowed determination of the K_M_ for Pdi1 (5.67 ± 2.36 μM) (Fig. 4b).

We hypothesized the Pdi1-independent step could be linked to the breakage of the intermolecular disulfide between Cys136 of Ero1 and Cys404 of Pdi1 in the Ero1-Pdi1 inactive complex. Resolution of this linkage is likely to be mediated by Cys407, the C-terminal active site cysteine of the *a’* domain. To test this hypothesis, we assayed the Ero1-Pdi1^C407A^ mutant complex with tenfold excess external Pdi1. Two activation steps were still observed, one of which was significantly delayed (approximately by three-fold) compared to the wild-type Ero1-Pdi1 complex (Table 1). This confirmed our hypothesis that the reduction of the mixed disulfide determines the kinetics of one of the two steps of activation. It is also consistent with our results showing that this mutation stabilized the complex by NEM-trapping SDS-PAGE, mass spectrometry and ThermoFluor.

Next, we analyzed the activity of monomeric Ero1^C136A^ with tenfold excess Pdi1. As expected, it showed only one activation step (Table 1), as it lacks the Cys136-Cys404 intermolecular disulfide. Both Ero1-Pdi1^C407A^ and Ero1^C136A^ mutations seemed to affect K_cat_, and the latter also the Hill coefficient for oxygen (Table 1).

To confirm that the second activation step of the wild-type complex was reduction of the Cys76-Cys332 intramolecular regulatory disulfide in Ero1, we attempted expression of the hyperactive Ero1^C76/332A^ mutant. Unfortunately, despite optimization, this could not be produced most likely due to severe toxicity effects.

### Effects of excess unfolded protein

The crosstalk between the oxidative folding machinery and the UPR pathways is still largely unexplored. In human cells, misfolded proteins or folding intermediates expose hydrophobic residues which are bound by the *b’* domain of hPDI^37^. This same domain interacts and recognizes Ero1α as a partner folding factor through its β-hairpin. Previous work conducted on hPDI and Ero1α highlighted how this single binding site for either a partner chaperone or a client substrate provides the conditions for a feedback inhibition loop, resulting in the complete arrest of oxygen consumption in the presence of unfolded proteins^22^. In *S. cerevisiae* Ero1*,* the β-hairpin is much shorter and does not expose highly hydrophobic residues to the environment. Correspondingly, in the AlphaFold-generated^32,33^ structure for *K. phaffii* Ero1 was also shorter and lacked the exposed tryptophan (Supplementary Fig. S7). This suggests that the β-hairpin either plays a less central role in the interaction with the *b’* domain in comparison to Ero1α, or in yeast the mechanism of recognition is handled differently. Moreover, it hints that the yeast system may not be regulated by an excess of unfolded substrate in the same way.

To examine this, we recreated the overburdening conditions in vitro by adding two model molecules to the Ero1 oxygen consumption assay set-up: reduced bovine pancreas trypsin inhibitor (BPTI) or the peptide KFWWFS. BPTI represents the ideal model to study refolding, as it is a small protein with three non-consecutive disulfide bonds in the native state^38,39^, while the KFWWFS peptide is a redox-insensitive synthetic peptide, which reproduces the hydrophobic core of unfolded proteins getting exposed to the solvent^37^. Both were previously proved to be strong inhibitors of the hPDI- Ero1α interaction^22,43^. BPTI was added in its reduced state during the catalysis at 50% residual oxygen, to avoid the generation of partially folded mixed disulfide intermediates before the start of the reaction. In contrast, the KFWWFS peptide was mixed with Pdi1 at the start of the assay, to be able to evaluate how the presence of the peptide would affect the activation phase.

**Figure 5:**
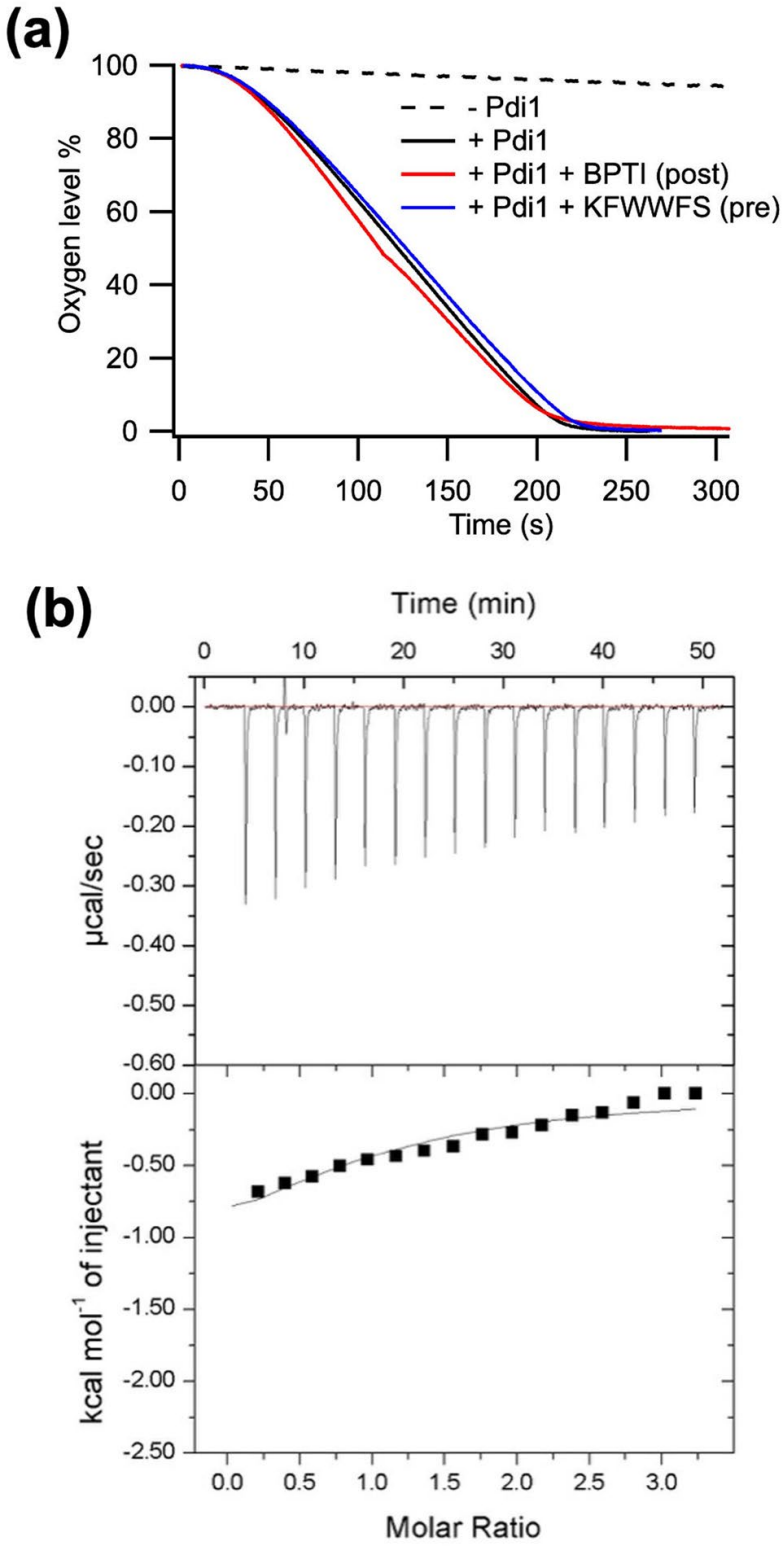
(**a**) Oxygen consumption traces of the Ero1-Pdi1 complex in the presence (positive control) and absence (negative control) of exogenous Pdi1, overlapped with traces including fivefold excess BPTI (injected at 50% residual oxygen) or fivefold excess KFWWFS peptide (present from the beginning of the assay). n = 3–5, one representative trace is shown; (**b**) ITC titration profile of 50 μM K*. phaffii* Pdi1 and 0–750 μM KFWWFS peptide.

Despite being in fivefold excess over the concentration of Pdi1, neither substrate generated any measurable effect on the kinetics (Fig. 5a) or turnover (both substrates) or activation (KFWWFS only), revealing an opposite outcome to the data previously reported on hPDI and Ero1α^22^. This either supports the idea that the yeast system differs from the human system and excess unfolded substrate does not inhibit Ero1-Pdi1 activity or that neither BPTI nor KFWWFS is bound by *K. phaffii* Pdi1.

As the second option could easily be verified, we first performed isothermal calorimetry (ITC). The binding profile showed extremely low affinity for KFWWFS, such that it was not possible to extrapolate accurate K_d_ values (Fig. 5b). Hence, hPDI and *K. phaffii* Pdi1 have different substrate binding specificities, and this explains the lack of inhibition by KFWWFS in both turnover and activation in the oxygen consumption assay by the *K. phaffii* enzymes.

To examine whether *K. phaffii* Pdi1 interacts with unfolded BPTI, we performed a classical Pdi refolding assay. BPTI refolding can be followed by a range of biophysical methods^40–42^: we employed a mass-spectrometry based assay^43^ to monitor the oxidation of BPTI from the completely reduced state (0S) through oxidative intermediate states (1S and 2S) to the native state (3S).

**Figure 6:**
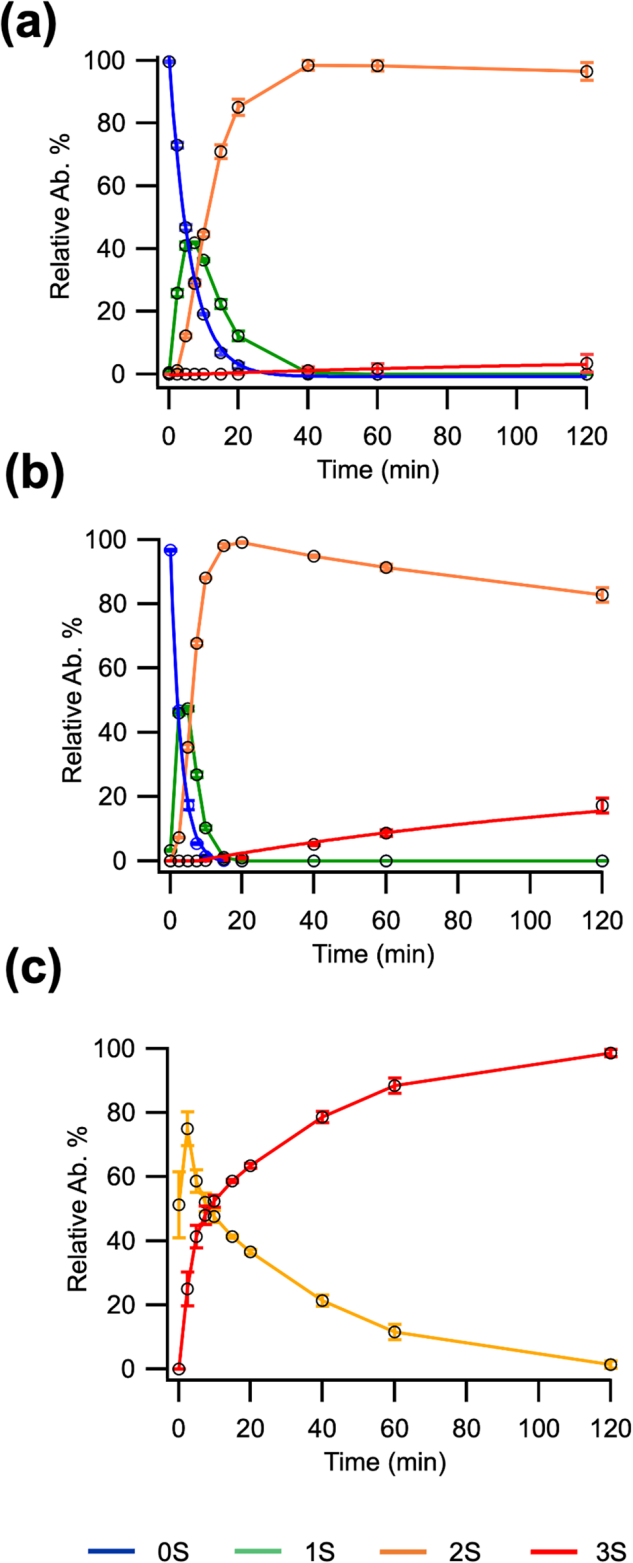
(**a**) Spontaneous BPTI oxidation (control reaction), (**b**) BPTI oxidation and (**c**) isomerization profile catalyzed by respectively 0.1 and 7 μM K*. phaffii* Pdi1. n = 3, data represented as average and standard deviation.

While spontaneous oxidation of BPTI was observed in the glutathione redox buffer (Fig. 6a), the reaction catalyzed in the presence of 0.1 μM K*. phaffii* Pdi1 (Fig. 6b) yielded significantly more native species (approximately fivefold more) at the final 2 h time point. However, isomerization of late-stage intermediates was too slow to be observed accurately in these conditions. Therefore, the reaction was repeated with 7 μM Pdi1. At the two-hour time-point, the native species could reach 100% and all 2S intermediates disappeared, indicating the reaction could complete (Fig. 6c).

It was concluded that *K. phaffii* Pdi1 can bind and refold BPTI, but that it does not have any affinity for the hydrophobic peptide. The ability to bind BPTI implies that the *K. phaffii* oxidative pathway is not inhibited by excess unfolded substrate and thus is regulated differently compared to the human system.

## Discussion

Disulfide bond formation in the ER is thought to be similar in all eukaryotic organisms, in terms of the enzymes involved, their mechanisms of action and regulation. Identically named enzymes across different species are often assumed to be synonymous. Since detailed mechanistic studies have only been conducted on folding factors from a very narrow range of organisms, this assumption of equivalence is potentially inexact. In fact, yeast species can be evolutionarily rather distant from each other, as for instance *K. phaffii* stands respectively to *S. cerevisiae*: both are budding yeasts, but *S. cerevisiae* underwent several additional losses and gains, including a whole genome duplication event^44^. Here we examined the main axis of oxidative folding in the ER of *K. phaffii*, the Ero1-Pdi1 pathway. *K. phaffii* Ero1 showed similarities and differences at the sequence and enzymatic level, when compared to its human and *S. cerevisiae* homologs. Firstly, the inactive state of *K. phaffii* Ero1 was a mixed disulfide state with Pdi1, with homologous cysteines involved in the intermolecular disulfide to human Ero1α/β and hPDI. A similar covalent complex has not been described for the *S. cerevisiae* enzyme, where the inactive state is thought to be monomeric Ero1^9, 45^. While the inactive complex is shared between the human and *K. phaffii* proteins, kinetics highlighted functional differences. Activation of the *K. phaffii* complex was faster (32 s) than for the human complexes (94 s and 63 s for α and β, respectively^13^, possibly due to the smaller number of regulatory disulfide bonds*.* K_cat_ values were similar between the *K. phaffii* and human enzymes, with the former showing slightly faster turnover: 1.2 s^−1^ for *K. phaffii* and 0.6 s^−1^ for Ero1α or 1 s^−1^ for Ero1β^13^. K_M_ values for PDI were also quite close: 5 μM for *K. phaffii* Pdi1 and 3 μM for hPDI with Ero1α, or 8 μM with Ero1β^13^. However, the Hill-coefficients for oxygen were much lower for the *K. phaffii* system (Hill = 2) compared with Ero1α (Hill = 5) and Ero1β (Hill = 3)^13^. This suggests that *K. phaffii* is much less adapted to hypoxic and hyper-hypoxic conditions, possibly due to the higher exposure that single cells experience as opposed to cells forming a tissue.

The most striking difference between the human and *K. phaffii* systems was the feedback inhibition by excess unfolded proteins. The human system is reported to be completely inhibited by excess unfolded proteins (or peptides), due to competition for binding by the *b’* domain of hPDI between Ero1α and folding substrates^22^. In stark contrast, no such inhibition was observed for the *K. phaffii* system, despite Pdi1 efficiently folding reduced BPTI to a native state, which indicates it must bind to the unfolded state of BPTI and to its intermediates. Therefore, the interaction site between Pdi1 and Ero1 must be different from that between Pdi1 and folding substrates. The protruding β-hairpin on Ero1α which is thought to interact with the *b’* domain in hPDI^22,37^ is much shorter in yeast enzymes and lacks the solvent-exposed tryptophan residue, which was shown to play a crucial role in this interaction^15^. Such structural difference implies that yeast Ero1 and Pdi1 rely much less on this *b’*-β-hairpin connection for their interaction and presumably make use of different recognition mechanisms, which have not been identified yet. One possible consequence of this difference is that shutdown of oxidative folding could be slower upon UPR activation in yeast, and hence cause a potentially slower recovery from the accumulation of unfolded proteins. The more protruding β-hairpin of human Ero1 that interacts with the same site of hPDI, which also binds to unfolded proteins, could represent an evolutionary gain of function in the human system over yeast, as it would allow to react faster to folding stress by immediately putting oxidative folding to a halt.

## Methods

### Vector assembly

Bacterial expression vectors were generated with classical restriction digestion. Protein coding genes were ordered codon optimized for *E.coli* from Twist Bioscience*,* while restriction sites were added by PCR*.* A pET23 backbone with a lactose inducible Tac promoter was used for the expression of all His-tagged proteins^46^. Cloning of polycistronic constructs was carried out at NdeI/BamHI sites for the first gene and SpeI/XhoI for the second. For monocistronic vectors, the combination of NdeI/XhoI was used. The customized version of CyDisCo was generated from pMJS226, a modified pLys vector housing the lacI repressor^47^. Human PDI was removed from the vector and substituted by *K. phaffii* Pdi1 by Gibson assembly (ClonExpress II cloning kit, Vazyme Biotech). *E. coli* XL1-blue (Agilent) was used as a propagation strain for all bacterial constructs. Cysteine-targeting point mutations were introduced by site-directed mutagenesis using standard protocols (QuickChange mutagenesis kit, Stratagene).

Yeast vectors were generated using Golden Gate cloning in the GoldenMOCS library adapted for *S. cerevisiae*^48^. Expression vectors comprised an Ars1 self-replicating origin, a Gal1 lactose inducible promoter controlling the expression of the coding sequence, followed by a Ura3 and a His3 cassette for auxotrophy complementation. The wild-type sequence of Ero1 was PCR amplified from the genome of *Komagataella phaffii* CBS7435. Cysteine-targeting point mutations were introduced by site-directed mutagenesis using standard protocols (QuickChange mutagenesis kit, Stratagene).

All plasmid constructs were sequenced prior to expression experiments.

### Spotting assays

*S. cerevisiae* BY4741 *ire1*Δ from the Euroscarf collection was transformed with the pArs-Cen constructs, each harboring different Cys to Ala mutations (single or in combination) of *K. phaffii* Ero1. Transformation was replicated according to the lithium acetate/PEG method described in Gietz and Schiestl^49^ and selection was carried out in yeast synthetic drop-out media supplemented with 60 mg/L leucine and 2% glucose. Single colonies were streaked twice on the same medium before subculturing in liquid drop-out media, 60 mg/L leucine and 2% raffinose. Liquid cultures were carried out in 24 deep-well plates, in 2 mL volume at 25 °C and 250 rpm shaking. After 24 h, OD_600_ was measured and levelled to 0.3 for all cell cultures with phosphate base. Serial factor 10 dilutions were made and 5 µL were spotted on a synthetic drop-out plate containing 60 mg/L leucine and 2% galactose acting both as inducer and carbon source. Spotting plates were poured from the same batch of media, and each displayed: one parental control expressing an empty vector, one replicate expressing wild-type *K. phaffii* Ero1 and four biological replicates expressing the single cysteine mutants.

### Protein expression and purification

Bacterial cytoplasmic expressions were performed in *E. coli* BL21(DE3) with the addition of the adapted CyDisCo folding factors. His-tagged Ero1 was expressed as the second gene of an operon with Pdi1, under the regulation of lactose induction. The additional copy of Pdi1 on the PET23 vector proved to be beneficial for covalent capturing of free Ero1 in the cell and purification of a homogeneous 1:1 complex. Only the 1–384 active core of Ero1 was isolated, excluding the C-terminal amphipathic helix, as previously performed for *S. cerevisiae* Ero1^6,9,34^, and the Cys460 unpaired thiol. For the purpose of large-scale purifications (250 mL per flask culture volumes), monomeric Ero1^C136A^ was expressed in the same set-up but as a monocistronic gene, while the extra copy of Pdi1 was maintained for the small-scale tests (2 mL culture volumes). His-tagged Pdi1 was expressed in *E. coli* BL21(DE3) in absence of CyDisCo. For both protein families, cells were precultured in LB broth + 0.4% glucose at 37 °C until OD_600_ values between 1 and 4 were reached.

For small-scale expression of mutants, main cultures were grown in a 24 deep-well plate format in 2 mL autoinduction medium (Formedium™) + 0.8% glycerol at 250 rpm shaking, at 20 °C for Ero1 and at 30 °C for Pdi1 alone. For large-scale productions, Ero1 (wild-type and mutant) was cultivated in several 250 mL flasks and Pdi1 in 1 L flasks (10% fill volume) and later pooled, giving total expression volumes of 500 mL. In all cases, preculture inoculum was kept to 1/100 of the main culture volume. 100 µg/mL ampicillin (for the target protein expression plasmid) and 35 µg/mL choramphenicol (for CyDisCo maintenance) were supplemented at all stages. Ero1 cultures were harvested at 48 h from inoculation in the autoinduction media, while for Pdi1 production time was limited to 24 h. Small-scale pellets were resuspended to the initial volume with 20 mM sodium-phosphate, 150 mM NaCl, pH 7.0 (phosphate base), mixed with 100 µg/L lysozyme and 20 µg/mL DNaseI, incubated at 30 °C for 15 min, lysed by freeze-thawing and cleared by centrifugation.

Purification (small-scale) was carried out on Poly-Prep columns (Biorad) with 0.4 mL bed volume of HisPur Cobalt Agarose resin (ThermoFischer Scientific) and 1 mL lysate, as described by Gaciarz et al*.*^50^, with a final elution step in 50 mM EDTA in phosphate base. Pellets from large-scale Ero1 expressions were resuspended to the original culture volume with phosphate base and 20 µg/mL DNaseI, lysed by sonication, cleared by centrifugation, and loaded on a pre-equilibrated 5 mL HisTrap HP column (Cytiva). Elution was carried out in 10 column volume (CV) imidazole gradient from 0 to 300 mM in phosphate base. Eluate was quickly buffer exchanged in 20 mM sodium phosphate pH 7.0 and loaded on a pre-equilibrated 6 mL Resource Q anion exchange column (Cytiva). Proteins were eluted from 100 to 300 mM NaCl at a very shallow gradient (0.5% increment per CV). Fractions were pooled, buffer exchanged in phosphate base and incubated in ice overnight in a 1:2 molar excess of FAD^+^. After reflavination, the mixture was further purified by size exclusion chromatography with a Superdex 200 16/600 HiLoad column (Cytiva) pre-equilibrated in phosphate base (also used for the activity assays). After SDS-PAGE visualization, the purest fractions were concentrated to 25 µM, aliquoted, flash-frozen in liquid nitrogen and stored at -70 °C. All purification steps were performed at 4° C. Monomeric Ero1 was purified following a similar procedure, with the omission of the anion exchange step in the middle.

The same steps were reproduced for the purification of Pdi1, except for the final reflavination and size exclusion, which were not needed in this case. Fractions deriving from anion exchange were visualized, the purest fractions were pooled, buffer exchanged in phosphate base, concentrated to 250 µM, flash-frozen and stored at -70 °C.

Protein concentration was determined with theoretical molecular masses, extinction coefficients and absorbance measurements at 280 nm. Given that Ero1 is a flavoenzyme, concentration of bound FAD^+^ was determined by absorbance at 454 nm (ε = 12,500 M^−1^ cm^−1^,^6^) and subtracted from the total protein concentration at 280 nm.

### SDS–PAGE and sample preparation

Reduced SDS–PAGE samples were prepared by mixing protein samples with loading buffer containing 1 M DTT, left incubating for 15 min at room temperature and heated up at 95 °C for 5 min. Non reduced samples were treated with NEM at the final concentration of 25 mM for 15 min, mixed with loading buffer without reducing agent and heated up at 95 °C for 5 min.

### Intact protein mass spectrometry

Purified protein samples were diluted to 0.1 mg/ml in 20 mM sodium phosphate pH 7.0 and either directly acidified with TFA (final concentration 0.1%) or pre-incubated for 10 min in 25 mM NEM both in the presence and absence of 4 M guanidium chloride as a denaturant. Samples were separated on a Waters BioResolve TMRP mAB polyphenyl column (450 Å, 2.7 µm, 2.1 × 50 mm) in 0.1–0.5% formic acid with increasing acetonitrile gradient and analyzed by electrospray ionization MS on a Q-Exactive Plus Orbitrap instrument.

### Circular dichroism

Circular dichroism (CD) spectra were recorded on a Chirascan-plus CD spectrophotometer (Applied Photophysics) between 280 and 185 nm at 22 °C in a 0.1 cm optical path quartz cuvette. CD measurements were acquired every 1 nm with 0.5 s integration time and repeated three times with baseline correction. Samples were measured in 10 mM sodium phosphate pH 7.0. Data was processed using Chirascan Pro-Data Viewer (Applied Photophysics).

### Thermostability

Thermostability curves were recorded in four replicates per sample on a ThermoFluor CFX96 thermocycler.

Purified protein samples were diluted to 0.5 mg/mL in phosphate base, mixed with SYPRO Orange protein stain (Thermo Fischer Scientific). Denaturation cycle was carried out between 10 and 95 °C in increments of 0.5 °C for 10 s. Triplicate curves were collected for all samples.

### Isothermal calorimetry

Protein–ligand interaction experiments were performed on a MicroCal iTC-200 (Malvern) calorimetry instrument. Pdi1 and KFWWFS peptide were diluted in phosphate base to the final concentrations of 50 and 750 µM respectively. The titration carried out at 25 °C with an initial injection of 0.4 µL followed by 2.44 µL peptide to protein injections at 3 min intervals. The data was analyzed using OriginLab. The first datapoint corresponding to the first injection was excluded from the final set, as advised by the manufacturer.

### Oxygen consumption assays

Activity of Ero1 was assessed by oxygen consumption with a Clark-type oxygen electrode (Oxytherm, Hansatech Ltd). Experiments were conducted essentially as described in Moilanen et al*.*^13^, with the addition of FAD^+^. Briefly, 1 µM of Ero1-PDI complex was injected into a reaction mixture containing 2 mM EDTA, 150 mM NaCl, 10 µM PDI, 50 µM FAD^+^ and 10 mM GSH at pH 7.0 in phosphate base. To keep the catalytic cycle active until oxygen would become the only limiting substrate in the assay, 1 mM NADPH and 0.05 U/µL glutathione reductase were added to the reaction mix. As a starting point, Pdi1 and GSH were incubated in the reaction chamber for 4 min, so that both active sites would get reduced, after which the Ero1-Pdi1 complex was injected. Reaction was recorded until oxygen levels were completely depleted. The control reaction without Pdi was recorded similarly but injecting 1 µM of Ero1^C136A^ mutant instead of the Ero1-Pdi1 complex. As a first step to analyze the activation dynamics, as well as determining the limiting concentrations to set-up the kinetic experiments, we performed a [GSH] titration while maintaining [Pdi1] unchanged to tenfold excess over the complex. We could thereafter determine that [GSH] 10 mM was not limiting to the reaction and that varying [GSH] would not impact either one of the activation rates (Supplementary Fig. S8). Inhibitors were added at 50 µM either before Ero1 was injected to the assay, or at approximately 50% oxygen saturation. Injection volumes of inhibitors were kept to less than 20 µL to avoid the risk of perturbations. Three to five traces were collected per reaction condition. Kinetic parameters such as K_cat_, activation rates, K_M_ and Hill coefficient were obtained with fitting functions adapted from Moilanen et al.^13^. The absolute limit of detection (LoD) for the oxygen consumption detection system is not known. The control unit nominally has a resolution limit of 0.0003% O_2_ and the electrode has a 10–90% response time of less than 5 s. Over the much smaller rate of oxygen changes we are measuring, the exact instrument response time is unknown. However, this type of oxygen electrode typically has a response time of the order under 1 s. As such, it is sufficiently fast as to not have an impact on the kinetic modeling under the conditions used. Higher concentrations of enzymes could not be examined due to potential limitations in the response time of the electrode.

### BPTI oxidation and refolding assays

Both assays were performed in a reaction buffer containing 0.5 mM GSSG, 2 mM GSH, 0.1 M phosphate and 1 mM EDTA at pH 7. A similar protocol as in Woehlbier et al*.* was employed. The oxidation reactions were carried out with 50 µM BPTI and 0.1 µM K*. phaffii* Pdi1, while isomerization was performed with 7 µM catalyst. Reactions were sampled at distinct intervals and rapidly quenched with 100 mM NEM, left incubating for 1 min, flash frozen in liquid nitrogen and stored at − 70 °C until analyzed by electrospray ionization MS. All reaction conditions were performed in triplicates.

### Supplementary Information

Supplementary Information.

## Data Availability

The data presented in this study is contained within the article and supplementary material S1. The datasets are available from the corresponding author on reasonable request.
