# Supplementary material for: Biochemical analysis of Komagataella phaffii oxidative folding proposes novel regulatory mechanisms of disulfide bond formation in yeast
Source: Sci Rep. 2023 Aug 31;13:14298. doi: 10.1038/s41598-023-41375-z (PMC10471769; doi:10.1038/s41598-023-41375-z)
Supplement: Supplementary file 1 — Supplementary Information. [file 41598_2023_41375_MOESM1_ESM.pdf]

## Supplementary Information

**Supplementary Figure S1**. Multiple sequence alignment of *K. phaffii* Ero1, *S. cerevisiae* Ero1, human Ero1α and Ero1β. Signal sequence is shown in underlined italics, while regulatory cysteines are highlighted in red, conserved active sites in yellow, cysteines of unknown or structural function in grey and cysteines involved in intermolecular disulfides with PDI in green.

```
Ero_alpha         -------MGRGWGF--LFGLLGAVWLLSSGH---GEEQPPETAAQRCFCQV---SGYLDD   45
Ero_beta          MSQGVRRAGAGQGVAAAVQLLVTL-----------------SFLRSVVEAQV---TGVLDD   41
K. phaffii        -----MRIVRSVAIAIACHCITALANPQ------------IPFD------GNYTEIIVPD   37
S.cerevisiae      -----MR----LRTAIATLCLTAFTSATSNNSYIATDQTQNAFNDTHFCKVDRNDHVSPS   51
                                      : :.                                   .

Ero_alpha         CTCDVETIDRFNNYRLFPRLQKLLESDYFRYYKVNLKRPCPFWND-ISQCGRRDCAVKPC  104
Ero_beta          CLCDIDSIDNFNTYKIFPKIKKLQERDYFRYYKVNLKRPCPFWAE-DGHCSIKDCHVEPC  100
K. phaffii        TEVNIGQIVDI-NHEIKPKLVELVNTDFFKYYKLNLWKPCPFWNGDEGFCKYKDCSVDFI   96
S.cerevisiae      CNVTFNELNAI-NENIRDDLSALLKSDFFKYFRLDLYKQCSFWDANDGLCLNRACSVDVV  110
                      .  :  : . .:   :  * : *:*:*::::* : * **    . *  : * *.

Ero_alpha         QSD-EVPDGIKSASY-KY----SEEANNLIEECEQAERLGAVDESLSEETQKAVLQWTKH  158
Ero_beta          PES-KIPVGIKAGHSNKYL--KMANNTKELEDCEQANKLGAINSTLSNQSKEAFIDWARY  157
K. phaffii        TDWSQVPDIWQPDQLGKLGDNTVHKDKGQDENE---------------------------  129
S.cerevisiae      EDWDTLPEYWQPEILGSFNNDTMKEADDSDDECKFLDQL-------CQTSKK-----PVD  158
                   .   :*   :     .       :     ::

Ero_alpha         DDSSDNFCEADDIQSPEAEYVDLLLNPERYTGYKGPDAWKIWNVIYEENCFKPQTIKRPL  218
Ero_beta          DDSRDHFCELDDERSPAAQYVDLLLNPERYTGYKGTSAWRVWNSIYEENCFKPRSVYRPL  217
K. phaffii        -LSSNDYCALDKDDDEDLVYVNLIDNPERFTGYGGQQSESIWTAVYDENCFQPNEG----  184
S.cerevisiae      IEDTINYCDVNDFNGKNAVLIDLTANPERFTGYGGKQAGQIWSTIYQDNCFTIGET----  214
                    .  .:*  :.  .     ::*  ****:*** * .:  :*. :*::***

Ero_alpha         NPLASGQGTSEENTFYSWLEGLCVEKRAFYRLISGLHASINVHLSARYLLQETWLEKKWG  278
Ero_beta          NPLAPSRGEDDGESFYTWLEGLCLEKRVFYKLISGLHASINLHLCANYLLEETWGKPSWG  277
K. phaffii        -------------SQLGQVEDLCLEKQIFYRLVSGLHSSISTHLTNEYLNLK---NGAYE  228
S.cerevisiae      -------------G-------ESLAKDAFYRLVSGFHASIGTHLSKEYLNTK---TGKWE  251
                                        .: *  **:*:**:*:**. **  .**  :      :

Ero_alpha         HNITEFQQRFDGILTEGEGPRRLKNLYFLYLIELRALSKVLPFFERPDFQ---LFTGNKI  335
Ero_beta          PNIKEFKHRFDPVETKGEGPRRLKNLYFLYLIELRALSKVAPYFERSIVD---LYTGNAE  334
K. phaffii        PNLKQFMIKV------GYFTERIQNLHLNYVLVLKSLIKLQEYNVIDNLPLDDSLKAGLS  282
S.cerevisiae      PNLDLFMARI------GNFPDRVTNMYFNYAVVAKALWKIQPYLPEF--SFCDLVN----  299
                   *:  *  :.      *    *: *::: * :  ::* *:  :            .
```

```
Ero_alpha         QDEENKMLLLEILHEIKSFPLHFDENSFFAGDKKEAHKLKEDFRLHFRNISRIMDCVGCF    395
Ero_beta          EDADTKTLLLNIFQDTKSFPMHFDEKSMFAGDKKGAKSLKEEFRLHFKNISRIMDCVGCD    394
K. phaffii        GLISQGAQ--GINQ--SSDDYLFNEKVLFQNDQ--NDDLKNEFRDKFRNVTRLMDCVHCE    336
S.cerevisiae      KEIKNKMD--NVIS--QLDTKIFNEDLVFANDL--SLTLKDEFRSRFKNVTKIMDCVQCD    353
                     .       :    .     *:*. .* .*      **::** :*:*::::**** *

Ero_alpha         KCRLWGKLQTQGLGTALKILFSEKLIANMPESGPSYEFHLTRQEIVSLFNAFGRISTSVK    455
Ero_beta          KCRLWGKLQTQGLGTALKILFSEKEIQKLPENSPSKGFQLTRQEIVALLNAFGRLSTSIR    454
K. phaffii        RCKLWGKLQTTGYGTALKILFDLKNPNDSI--------NLKRVELVALVNTFHRLSKSVE    388
S.cerevisiae      RCRLWGKIQTTGYATALKILFEINDADEFTKQ--HIVGKLTKYELIALLQTFGRLSESIE    411
                  :*:****:** * .*******. :   .          :*.: *:::*.::* *:* *:.

Ero_alpha         ELENFRNLLQNIH-----------------------------------------------    468
Ero_beta          DLQNFKVLLQHSR-----------------------------------------------    467
K. phaffii        SIENFEKLYKIQPPTQDRASASSESLGLFDNEDEQNLLNSFSVDQAVISSKEAPEE---I    445
S.cerevisiae      SVNMFEKMYGKRLNGSENRLSSFFQNNFFNILKEAGKSIRYTIENI-NSTKEGKKKTNNS    470
                  .:: *. :

Ero_alpha         ------------------------------------------------------------    468
Ero_beta          ------------------------------------------------------------    467
K. phaffii        KSKPVGK---AAYKQNSCPSLG-SKSIKEAFHEELHAFIDAIGFILNSYRTLPKLLYTLF    501
S.cerevisiae      QSHVFDDLKMPKAEIVPRPSNGTVNKWKKAWNTEVNNVLEAFRFIYRSYLDLPRNIWELS    530


Ero_alpha         ---------------------------------  468
Ero_beta          ---------------------------------  467
K. phaffii        LVKSSELWDIFIGTQRHRDT------TYRVDL-  527
S.cerevisiae      LMKVYKFWNKFIGVADYVSEETREPISYKLDIQ  563
```

**Supplementary Figure S2.** Spotting assay of *S. cerevisiae* BY4741 *ire1*Δ overexpressing *K. phaffii* Ero1 Cys136Ala, Cys178Ala, Cys194Ala and Cys460Ala. WT= wild-type Ero1, - = empty vector control.

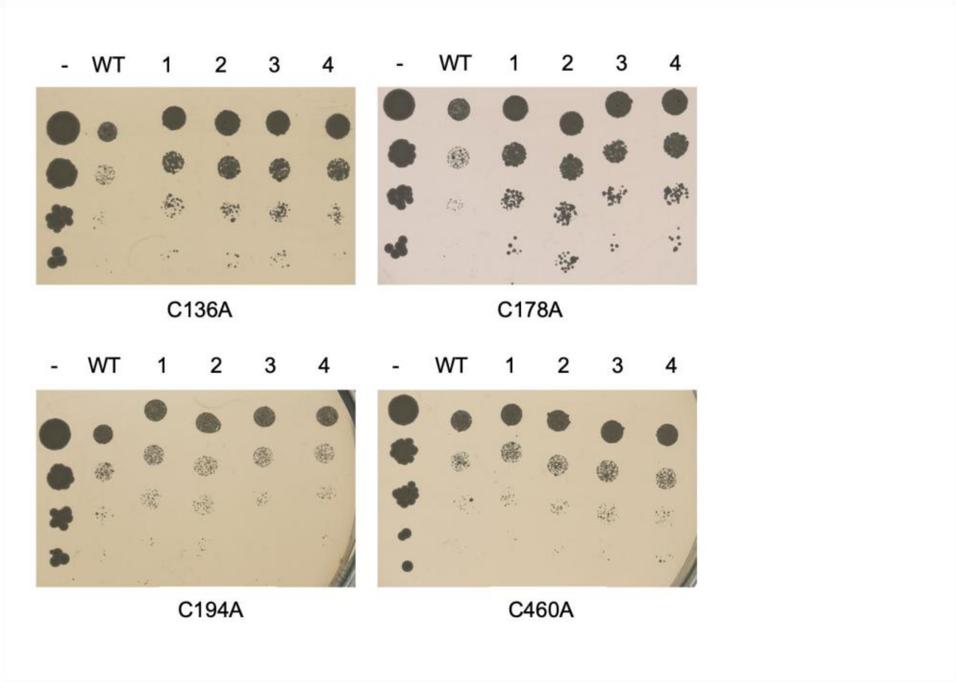

**Supplementary Figure S3.** AlphaFold predicted structure *of K. phaffii* Ero1 (in yellow), cysteines are displayed in orange, while Cys136 is highlighted in red. Zoomed area displays predicted *K. phaffii* Ero1 (in yellow), aligned to Ero1α (in magenta, PDB ID: 3AHR) and *S. cerevisiae* Ero1 (in light blue, PDB ID: 1RP4). *K. phaffii* Ero1 Cys136 is shown to be close to approximate location of Ero1α Cys166 (residue not present in the crystal structure, replaced by red dashed line).

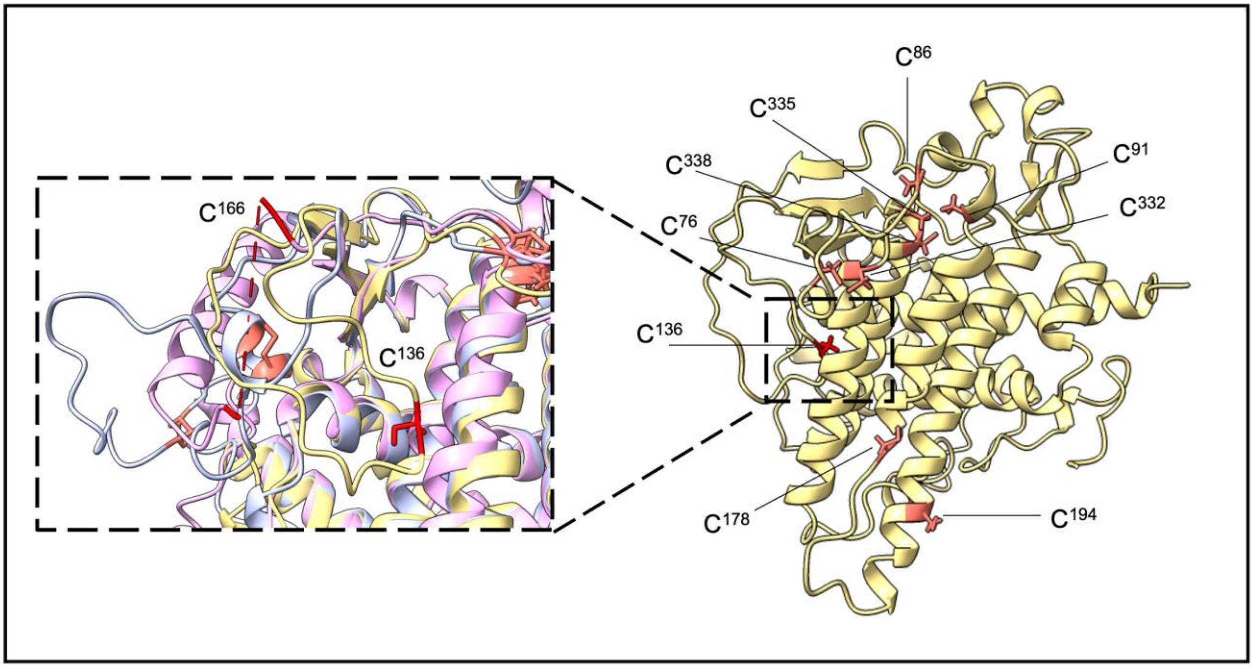

**Supplementary Figure S4.** Full uncropped image of SDS-PAGE gel showed in Figure 1.

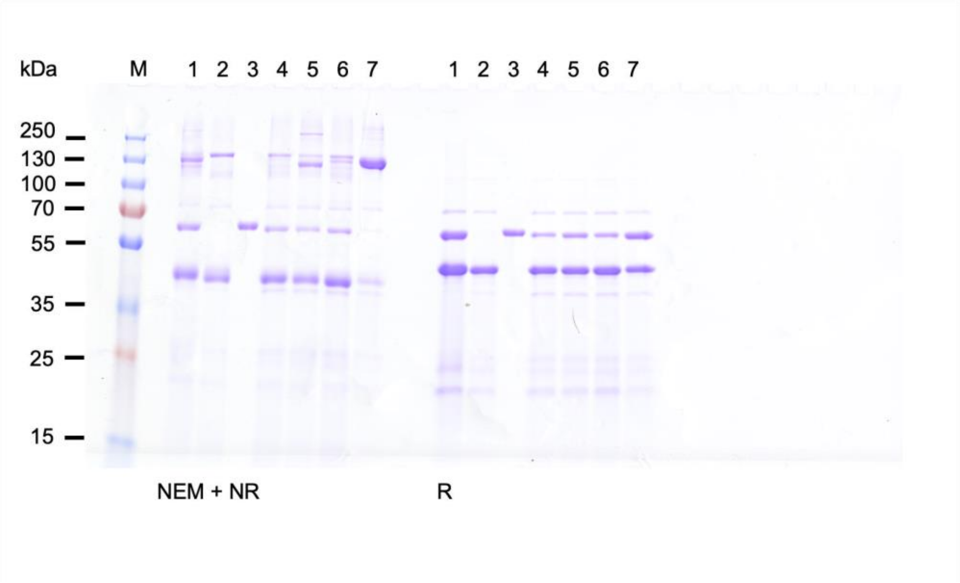

**Supplementary Figure S5.** a) Mean residue molar ellipticity spectra of *K. phaffii* Pdi1 and b) wild-type *K. phaffii* Ero1-Pdi1 reflavinated (red) and non-reflavinated (black).

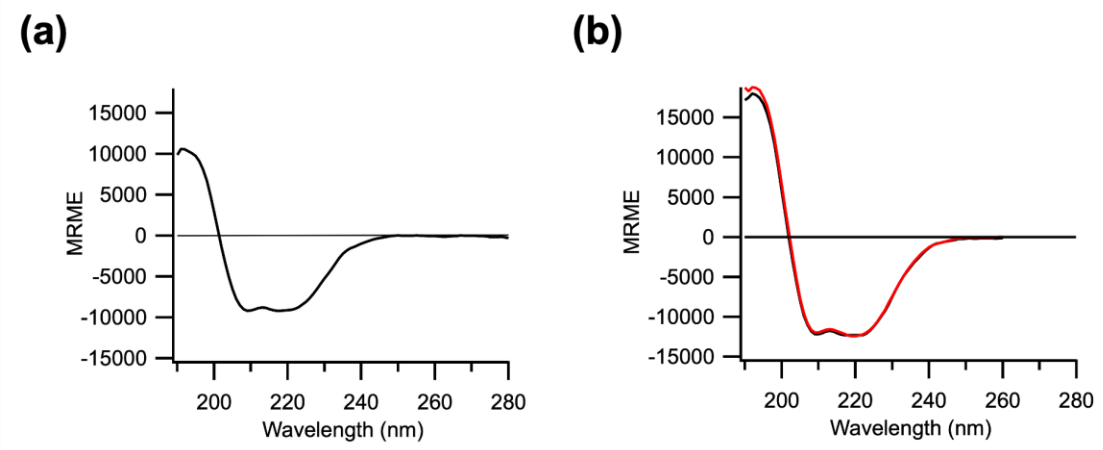

**Supplementary Figure S6.** a) RFU derivative curves over temperature of *K. phaffii* Pdi1 and b) wild-type *K. phaffii* Ero1-Pdi1 reflavinated (red) and non-reflavinated (black).

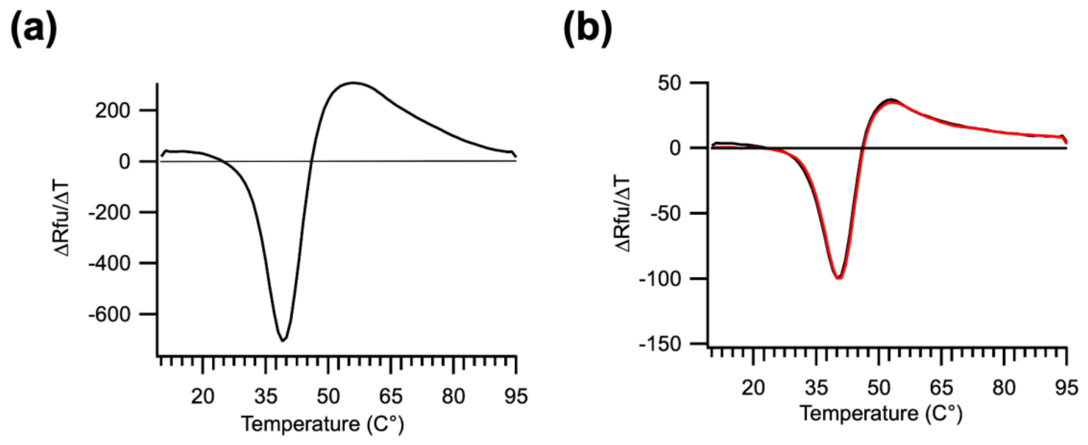

**Supplementary Figure S7.** Structural alignment of b-hairpin on Ero1α (in magenta, PDB ID: 3AHR), where $Trp^{272}$ is highlighted, *K. phaffii* Ero1 (in yellow, AlphaFold-generated model), *S. cerevisiae* Ero1 (in light blue, PDB ID: 1RP4). Displayed sidechains of residues composing the β-hairpin: *K. phaffii* Ero1 $L^{220}$ $K^{221}$ $N^{222}$; *S. cerevisiae* Ero1 $T^{245}$ $K^{246}$ $T^{247}$; Ero1α $W^{272}$ $L^{273}$ $E^{274}$.

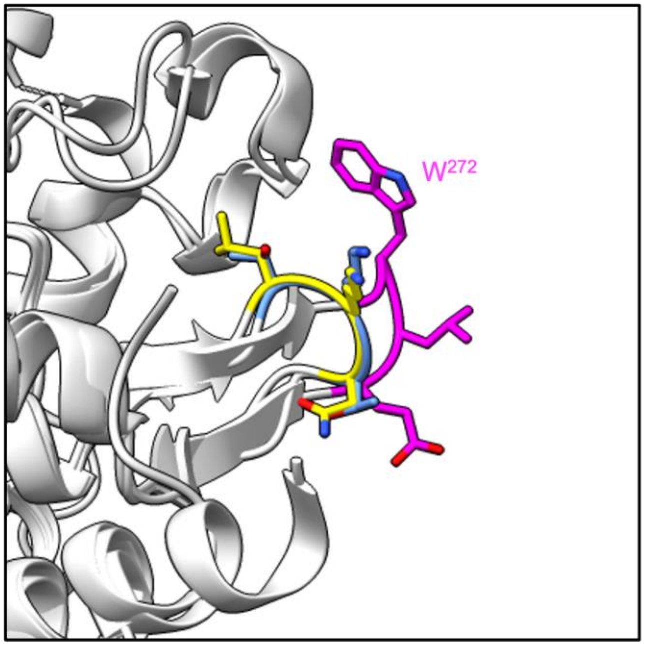

**Supplementary Figure S8.** Effect of GSH titration on (a) activation rates (first rate, open squares □; second rate, closed hexagons ⬢) and (b) $K_{cat}$ with 10 µM Pdi1 and 1 µM Ero1-Pdi1 wild-type complex.

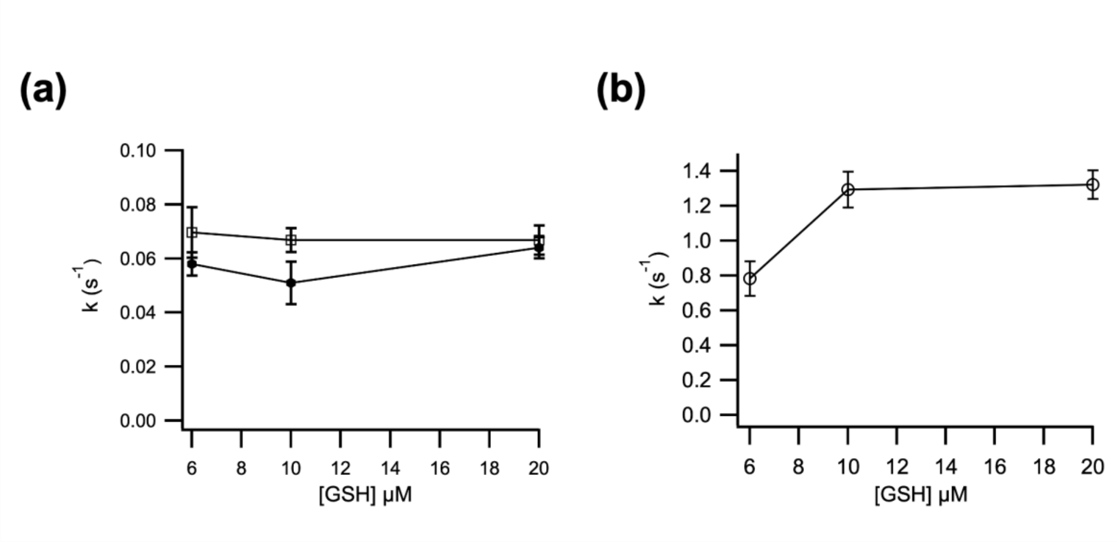

**Supplementary Tables**

**Supplementary Table S1.** Electrospray ionization mass spectrometry analysis of purified complexed and monomeric Ero1 and Pdi1. Both theoretical and experimental masses are reported as average masses. Theoretical average masses refer to the oxidized state of the proteins. *= cleavage of initiating methionine on Pdi1 was consistently observed. The theoretical mass for Pdi1 is reported without the initiating methionine.

| NEM | Protein | Theoretical (Da) | Experimental (Da) | Disulfides | Δ |
|---|---|---|---|---|---|
| - | Pdi1_6His | 56345.89 * | 56345.00 | 3 | 0.89 |
| | Ero1_C136A_6His | 45087.63 | 45086.97 | 4 | 0.66 |
| | Ero1_6His-Pdi1<br>Pdi1<br>Ero1_6His | 100496.80 *<br>55379.110*<br>45119.69 | 100496.35<br>-<br>- | 7<br>3<br>4 | 0.45<br>-<br>- |
| | Ero1_6His-<br>Pdi_C407A | 100466.74 * | 100466.54 | 6 | 0.20 |
| + | Pdi1_6His | 56345.89 * | 56344.80 | 3 | 1.09 |
| | Ero1_C136A<br>_6His | 45087.63 | 45087.26 | 4 | 0.37 |
| | Ero1_6His-Pdi1<br>Pdi1<br>Ero1_6His | 100496.80 *<br>55379.110*<br>45119.69 | -<br>55377.84<br>45243.65 | 7<br>3<br>4 | -<br>1.27<br>-123.96 (1 NEM) |
| | Ero1_6His-<br>Pdi1_C407A | 100466.74 * | 100465.48 | 6 | 1.26 |

**Supplementary Table S2**. List of plasmids used in this study

| Plasmid | Background | Promoter | Expressed Gene |
|---|---|---|---|
| pAR_EC1 | pET23 | tac | KpPdi1_6His |
| pAR_EC15 | pLys | tac | Erv1p_KpPdi1 |
| pAR_EC35 | pET23 | tac | KpEro1_6His |
| pAR_EC37 | pET23 | tac | KpEro1_Cys136Ala_6His |
| pAR_EC65 | pET23 | tac | KpPdi1_Cys65Ala_ KpEro1_6His |
| pAR_EC66 | pET23 | tac | KpPdi1_Cys407Ala_ KpEro1_6His |
| pAR_EC67 | pET23 | tac | KpPdi1_Cys62Ala_KpEro1_6His |
| pAR_EC68 | pET23 | tac | KpPdi1_Cys402Ala_ KpEro1_6His |
| pAR102 | Ars1-Cen | gal1 | KpEro1 |
| pAR91 | Ars1-Cen | gal1 | KpEro1_Cys13Ala |
| pAR92 | Ars1-Cen | gal1 | KpEro1_Cys15Ala |
| pAR93 | Ars1-Cen | gal1 | KpEro1_Cys76Ala |
| pAR94 | Ars1-Cen | gal1 | KpEro1_Cys136Ala |
| pAR95 | Ars1-Cen | gal1 | KpEro1_Cys178Ala |
| pAR96 | Ars1-Cen | gal1 | KpEro1_Cys194Ala |
| pAR97 | Ars1-Cen | gal1 | KpEro1_Cys332Ala |
| pAR98 | Ars1-Cen | gal1 | KpEro1_Cys460Ala |
| pAR108 | Ars1-Cen | gal1 | KpEro1_Cys76/332Ala |
